# Serotonin transporter: a potential new immune checkpoint for cytotoxic T cells

**DOI:** 10.1038/s41392-025-02449-8

**Published:** 2025-10-29

**Authors:** Gerard P. Ahern, Rosa L. Miyares

**Affiliations:** Department of Pharmacology and Physiology, Georgetown University, Washington, DC USA

**Keywords:** Immunotherapy, Adaptive immunity

In a recent publication in *Cell*, Yang and colleagues propose a novel role for the serotonin transporter (SERT) as an immune checkpoint in CD8^+^ T cells.^1^ These findings highlight the therapeutic potential of selective serotonin reuptake inhibitors (SSRIs) as anti-tumor drugs.

Serotonin (5-hydroxytrptamine, 5-HT) is a monoamine transmitter with various important roles in the body, including in the immune system. 5-HT signaling can promote immune cell migration and regulate cytokine production.^2^ Immune cells express a diverse repertoire of 5-HT receptors (5-HTR) on their cell surface. While all immune cells respond to exogenous 5-HT, T lymphocytes (in particular CD8^+^ or cytotoxic T cells) also produce serotonin. Indeed, T cells possess the complete machinery for 5-HT synthesis, storage, degradation, and secretion.^3^ It has been demonstrated in vitro that during T-cell receptor (TCR) signaling, the synthesis and release of 5-HT increases.^2^ This leads to autocrine stimulation of 5-HTRs and enhanced T-cell activation.^2^ Thus, serotonin appears to act as an accessory signal to help stimulate CD8^+^ T cells.^2,3^

Li et al. propose that SERT functions as a novel immune checkpoint in CD8^+^ T cells.^1^ Checkpoints evolved as a homeostatic mechanism to constrain an overactive immune response. However, these signals can also be exploited (enhanced) by cancer cells to suppress immune function. Li et al. report upregulated SERT in tumor-infiltrating CD8^+^ T cells compared with naïve cells, suggesting the tumor microenvironment alters T-cell serotonin signaling. Treatment with SSRIs, fluoxetine and citalopram, increased the number of intratumor CD8^+^ T cells and enhanced the cytotoxic functionality of these cells. Crucially, SSRI treatment also led to smaller tumor sizes and prolonged survival in both mouse syngeneic and human xenograft tumor models. SERT KO mice showed similar results as the SSRI treatments, supporting SERT as the SSRI target. Other cells, including cancer cells themselves, express SERT. Therefore, to identify a specific role for CD8^+^ T cells, Li et al. performed adoptive transfer experiments, transferring either normal or SERT-deficient immune systems/CD8^+^ T cells into recipient mice with irradiated immune systems. Once again, disrupting SERT in these mouse models led to enhanced CD8^+^ T-cell function (less cell exhaustion and greater killing capacity) and a smaller tumor size. To identify a cell-autonomous effect, they turned to cell culture experiments. In response to TCR stimulation, SERT KO CD8^+^ cells exhibited greater proliferation, cytokine generation, and cytotoxic function compared with wild-type CD8^+^ T cells. Similar results were obtained when wild-type CD8^+^ cells were stimulated in the presence of the SSRI, fluoxetine.

**Fig. 1:**
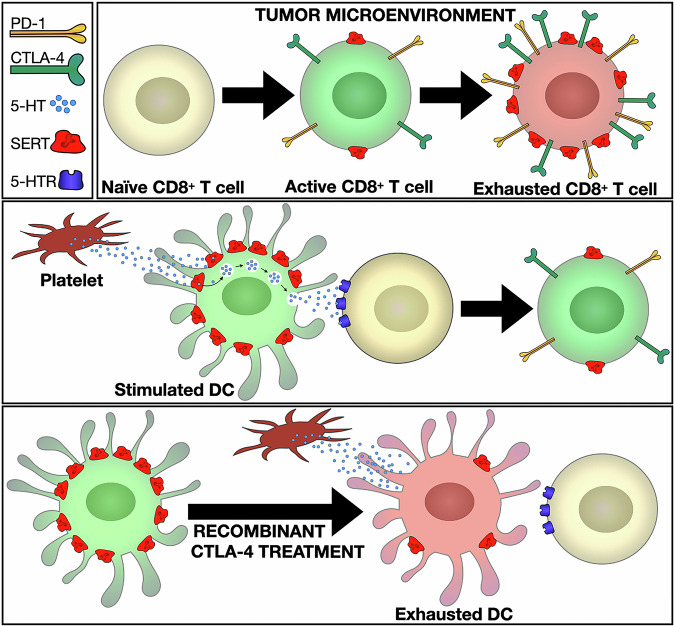
SERT, a common immune checkpoint signal. (*Top*) SERT is upregulated during CD8^+^ T-cell checkpoint activation. In tumor-infiltrating murine CD8^+^ T cells, elevated SERT levels correlate with high PD-1 expression,^1^ a marker of exhausted T cells. (*Middle*) Dendritic cells (DCs) enhance T-cell activation via serotonin signaling. Stimulated murine DCs have high SERT expression, allowing them to take up and secrete 5-HT^2^. During TCR activation, 5-HT secretion enhances T-cell activation.^2^ (*Bottom*) SERT is downregulated during DC checkpoint activation.^2^ Recombinant CTLA-4 treatment decreases SERT expression, hindering T-cell activation.^2^

SERT may function as a checkpoint in various immune cells. Checkpoint signaling in T cells through programmed death receptor 1 (PD-1) and cytotoxic T-lymphocyte-associated protein 4 (CTLA-4) is normally induced following T-cell activation. Interestingly, Li et al. find elevated SERT in mouse CD8^+^ T cells with high levels of PD-1, suggesting that SERT expression is enhanced by checkpoint signaling (Fig. 1). Interestingly, a previous study showed that immune checkpoint signaling regulates SERT expression in dendritic cells (DC, professional antigen-presenting cells). DCs also utilize serotonin signaling. At sites of inflammation, DCs take up 5-HT via SERT then subsequently secrete this 5-HT to T cells,^3^ enhancing the T-cell activation response (Fig. 1). The checkpoint signal, CTLA-4 suppresses DC SERT expression,^3^ thereby potentially weakening the DC’s ability to stimulate T cells (Fig. 1). Thus, checkpoint signaling produces opposing regulation of SERT expression in murine T cells and DCs, yet both have the same predicted outcome of inhibiting T-cell activation.

SSRI treatment likely affects tumor-associated serotonin signaling in various ways. Li et al. propose that SSRIs raise extracellular serotonin by inhibiting T-cell-expressed SERT; they believe that this results in enhanced autocrine signaling via 5-HTRs, which consequently activates CD8^+^ T cells. Just like in neurons, immune-cell SERT takes up 5-HT very effectively, with half-maximal transport attained at ~85 nM^2^. Thus, SERT may efficiently reduce the extracellular levels of 5H-T proximate to CD8^+^ T cells. Indeed, Li et al. showed that treatment with selective SSRIs elevated 5-HT levels within tumors, and this was abrogated after T-cell depletion. Further, in cell culture using serotonin-depleted media, the authors showed that SSRIs enhanced 5-HTR signaling and early T-cell activation markers. While these data support a T-cell autocrine signaling pathway, they do not exclude additional mechanisms. For example, SSRI treatment blocks storage of 5-HT in platelets, leading to reduced serum/tissue levels of 5-HT.^4,5^ In turn, the reduction in intratumoral 5-HT levels can decrease expression of tumor checkpoint signaling factors (e.g., PD-LI), thus enhancing the cytotoxic potential of CD8^+^ T cells.^4^ Further, Dong et al. showed that the SSRI, citalopram, directly interacts with complement component 5a receptor 1 (C5aR1) to stimulate phagocytosis in tumor-associated macrophages, leading to increased CD8+ T-cell immunity.^5^ Moreover, SSRIs given at high doses in vitro can directly induce tumor cell apoptosis.^2^ Thus, both SERT-dependent and SERT-independent mechanisms may contribute to the tumor-clearing actions of SSRIs.

While Li et al. observed differences between murine models and human cancer, SERT holds promise as a therapeutic target.^1^ In mouse CD8^+^ T cells, PD-1 and SERT expression levels correlated. However, analysis of human cancer RNAseq databases showed PD-1 and SERT expression in distinct cell populations. Exhausted human CD8^+^ T cells with high levels of classical checkpoint genes exhibited very low expression of SERT and other 5-HT signaling genes. In contrast, the most potent cytotoxic CD8^+^ T cells (terminally differentiated effector memory CD45RA re-expressing cells) exhibited very high levels of SERT. This may reflect differential regulation of SERT in human CD8^+^ T cells or, as the authors propose, high SERT may act as a brake, ultimately contributing to exhaustion of this population. Regardless, the culture of human CD8^+^ T cells in the presence of SSRIs resulted in an increase of serotonergic and T-cell effector gene expression, as well as an increase in cell count—similar to the mouse models. Further, analysis of clinical data showed a negative correlation between tumor *SERT* gene expression and patient survival. Moreover, Li et al. showed that SSRIs have the potential to enhance immune checkpoint therapy.^1^ In mouse models, SSRIs activated cell-signaling pathways in CD8^+^ T cells distinct from anti-PD-1 treatment. SSRIs synergized with anti-PD-1 therapy in tumor killing and effectively killed tumors unresponsive to anti-PD-1 treatment.

In summary, the very interesting findings by Li et al. add to the intricate and complex role of 5-HT in regulating the immune system. Although further studies are needed to optimize therapeutic potential, in the near future, SSRI treatment may become an additional quiver in the bow of anti-tumor therapy.
